# The Community Navigator Study: a feasibility randomised controlled trial of an intervention to increase community connections and reduce loneliness for people with complex anxiety or depression

**DOI:** 10.1186/s13063-017-2226-7

**Published:** 2017-10-23

**Authors:** Brynmor Lloyd-Evans, Jessica K. Bone, Vanessa Pinfold, Glyn Lewis, Jo Billings, Johanna Frerichs, Kate Fullarton, Rebecca Jones, Sonia Johnson

**Affiliations:** 1 Division of Psychiatry, University College London, London, W1T 7NF UK; 2 The McPin Foundation, London, SE1 0EH UK; 3 Camden and Islington NHS Foundation Trust, London, NW1 0PE UK

**Keywords:** Loneliness, Depression, Anxiety, Feasibility Study, Randomised controlled trial, Community navigation

## Abstract

**Background:**

Loneliness is associated with poor health outcomes at all ages, including shorter life expectancy and greater risk of developing depression. People with mental health problems are particularly vulnerable to loneliness and, for those with anxiety or depression, loneliness is associated with poorer outcomes. Interventions which support people to utilise existing networks and access new social contact are advocated in policy but there is little evidence regarding their effectiveness. People with mental health problems have potential to benefit from interventions to reduce loneliness, but evidence is needed regarding their feasibility, acceptability and outcomes. An intervention to reduce loneliness for people with anxiety or depression treated in secondary mental health services was developed for this study, which will test the feasibility and acceptability of delivering and evaluating it through a randomised controlled trial.

**Methods:**

In this feasibility trial, 40 participants with anxiety or depression will be recruited through two secondary mental health services in London and randomised to an intervention (*n* = 30) or control group (*n* = 10). The control group will receive standard care and written information about local community resources. The coproduced intervention, developed in this study, includes up to ten sessions with a ‘Community Navigator’ over a 6-month period. Community Navigators will work with people individually to increase involvement in social activities, with the aim of reducing feelings of loneliness. Data will be collected at baseline and at 6-month follow-up – the end of the intervention period. The acceptability of the intervention and feasibility of participant recruitment and retention will be assessed. Potential primary and secondary outcomes for a future definitive trial will be completed to assess response and completeness, including measures of loneliness, depression and anxiety. Qualitative interviews with participants, staff and other stakeholders will explore experiences of Community Navigator support, the mechanisms by which it may have its effects and suggestions for improving the programme.

**Discussion:**

Our trial will provide preliminary evidence of the feasibility and acceptability of Community Navigator support and of trial procedures for testing this. The results will inform a future definitive randomised controlled trial of this intervention.

**Trial registration:**

ISRCTN10771821. Registered on 3 April 2017.

**Electronic supplementary material:**

The online version of this article (doi:10.1186/s13063-017-2226-7) contains supplementary material, which is available to authorized users.

## Background

Loneliness has been defined as a subjective unpleasant feeling arising from a discrepancy between people’s desired and achieved levels of meaningful social relationships [1]. It is related to, and overlaps with, a range of concepts including social isolation, social capital, social network and social support [2]. Although related to objective social isolation, loneliness is a distinct subjective experience which may be driven by the quality as well as quantity of social relationships.

Prolonged loneliness is increasingly recognised internationally as a major public health issue [3–5]. It is distressing in itself and has been demonstrated to predict a range of poor health outcomes in the general population, including shorter life expectancy [6], elevated blood pressure [7], diminished immunity [8], and cognitive decline [9]. Loneliness predicts the onset of anxiety [10] and depression [11].

While public policy has focused primarily on initiatives to alleviate loneliness in older adults [12, 13], there is a growing recognition that these may need to be extended to people of all ages. In the UK, 6% of adults report being lonely all or most of the time [14] and 21% report being ‘sometimes’ lonely [15]. People with mental health problems typically have smaller social networks than the general population [16]. Up to 40% of people with depression feel lonely most of the time [15], and a tenfold increase in the odds of being lonely has been reported, compared to the general population [17]. For people with anxiety and depression, loneliness independently predicts poorer symptom outcomes 1 year later [18]. Interventions which alleviate loneliness for people with anxiety and depression, therefore, potentially promise not only improved quality of life, but also reduced mental health problems and less risk of a range of poor health outcomes.

There are a range of related social interventions which have the potential to alleviate loneliness and reduce social isolation. Interventions can be classified as ‘direct’, explicitly targeting loneliness and social relationships, or ‘indirect’ broader approaches to improving health and wellbeing that may have impacts for loneliness [19]. There are several types of direct intervention which aim to reduce loneliness including: changing people’s cognitions about social relationships; social skills training and psychoeducation about the value of social connections; supported socialisation; and wider community approaches to reducing loneliness [19].

Interventions involving supported socialisation are currently being widely adopted and appear to be particularly promising. These can include social prescribing – the provision of groups and/or financial assistance to attend groups which promote social integration or wellbeing [13]. Interventions can also involve brief individual support from a ‘navigator’ or ‘wellbeing coach’ for people to utilise existing networks and access new social contact and support in their local community [13, 19]. Social prescribing schemes have demonstrated that these approaches can be feasible and engaging in a mental health context. For example, Wellbeing Enterprises Community Interest Company (CIC) has been commissioned to provide social prescribing for people referred from primary care for over ten years. Routine evaluations suggest improvements in wellbeing and depression following receipt of their brief intervention [20].

Social interventions such as this are advocated in research literature and policy to provide integrated health and social care and improve health outcomes for people with long-term conditions, including mental health problems [21–23]. Although they have been reported positively, there is little evidence regarding their effectiveness for people with mental health problems [24]. It is also unclear whether such interventions, most commonly provided in primary care, are appropriate for a population with enduring mental health problems and complex needs in secondary care, who may have even greater problems with loneliness.

The aim of this study is to develop and test the feasibility and acceptability of a programme of support for people with complex depression and anxiety and to examine the feasibility of a randomised controlled trial (RCT) of this intervention. This programme will include receiving support from a ‘Community Navigator’ based in secondary mental health services, who will help service users to increase social contact, participation in social activities and community engagement, with the aim of reducing feelings of loneliness. The study comprises modelling, preliminary testing and a feasibility trial of the programme, in accordance with guidance for developing and evaluating a complex intervention [25]. This protocol is for the feasibility RCT of the intervention with mixed-methods evaluation.

### Aims

The main aims of the Community Navigator Feasibility RCT are:

1. To develop and manualise a social intervention involving Community Navigator support to increase social connections and reduce loneliness for people with complex depression and anxiety using secondary mental health services
2. To trial the programme with 40 service users to test acceptability of the intervention and trial procedures (including outcome measures and feasibility of participant recruitment and retention) and investigate optimal trial processes
3. To explore stakeholders’ experience of the programme, barriers and facilitators to its successful implementation, potential mechanisms of its effect, and refine a theory of change model which outlines the processes by which the programme may have an effect

## Methods

### Design

The study is a feasibility trial with block randomisation conducted in two sites in London (United Kingdom). Randomisation will be stratified by site with an allocation ratio of 3:1, intervention to control. The trial compares Community Navigation, a co-produced, newly developed social intervention provided in addition to standard care from secondary mental health services, to a control group receiving standard care and written information about local community resources.

**Fig. 1:**
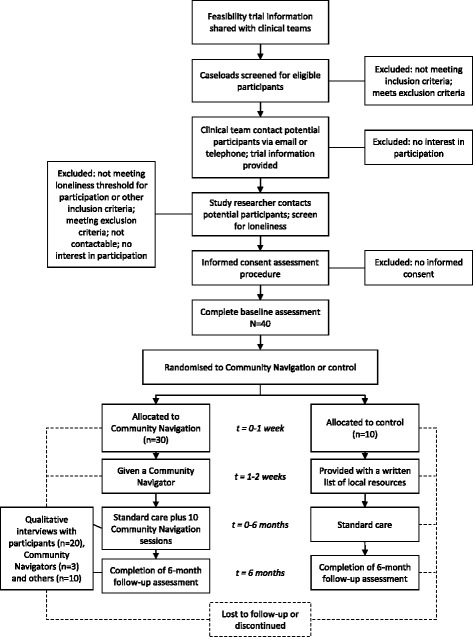
SPIRIT flow diagram of the phases of the Community Navigator Feasibility Trial

The design as described here adheres to the Standard Protocol Items: Recommendations for Interventional Trials (SPIRIT) guidelines [26], including a flow diagram (Fig. 1) and a SPIRIT schedule (Table 1). A copy of the SPIRIT Checklist, detailing where each recommended element of the protocol is included in this paper, is provided as Additional file 1.

**Table 1:** SPIRIT schedule of enrolment, interventions, and assessments for participants

	Study period			
Timepoint	Enrolment	Allocation	Treatment period (6 months)	Follow-up (end of treatment period; 6 months)
Enrolment:				
Eligibility screen	X			
Informed consent	X			
Randomisation		X		
Intervention:				
Community Navigation			X	
Control			X	
Assessments:				
Baseline outcome measures	X			
Sociodemographic characteristics				
Trust Informatics information				
De Jong Gierveld Loneliness Scale				
Lubben Social Network Scale				
Resource Generator UK				
Time Budget Diary				
WEMWBS				
Patient Health Questionnaire				
Generalised Anxiety Disorder Questionnaire				
EQ-5D-5 L				
Recovering Quality of Life Questionnaire				
Process recording			X	
Community Navigator session logs				
Participant feedback phone calls				
Follow-up outcome measures				X
Sociodemographic characteristics				
Trust Informatics information				
De Jong Gierveld Loneliness Scale				
Lubben Social Network Scale				
Resource Generator UK				
Time Budget Diary				
WEMWBS				
Patient Health Questionnaire				
Generalized Anxiety Disorder Questionnaire				
EQ-5D-5 L				
Recovering Quality of Life Questionnaire				
Qualitative interview			X	X

*WEMWBS* Warwick-Edinburgh Mental Well-being Scale, *EQ-5D-5 L* EuroQol 5-dimension, 5-level health outcome measure

### Setting

We will involve participants from two NHS sites: the Complex Depression, Anxiety and Trauma (CDAT) Team within Camden and Islington NHS Foundation Trust and the Mood, Anxiety and Personality stream of Barnet Complex Care Team (CCT) in Barnet, Enfield and Haringey Mental Health NHS Trust. Both services support about 600 adult service users with moderate or severe depression, anxiety or other affective disorders. Both services offer care coordination, support from a multi-disciplinary team, and psychiatric outpatient appointments. Neither service offers Community Navigation support as a standard part of care delivered by the team. In both areas, service users may access a range of statutory and voluntary sector services which provide various activities and opportunities for social contact. Both the inner-London boroughs of Camden and Islington and the outer-London borough of Barnet include affluent areas and areas of high deprivation, and ethnically diverse populations.

### Participants

At each study site, 20 service user participants will be recruited at baseline (total *N* = 40). This sample size has been chosen to be sufficient to give an indication of the acceptability of the Community Navigator programme and the feasibility of trial recruitment procedures. The treatment group will consist of 30 participants and the remaining 10 participants will form the control group.

To ensure that we are working with a population who are experiencing loneliness, we will use the six-item De Jong Gierveld Loneliness Scale [27] as a screening measure, for which a score of 2 has been established as a minimum threshold for loneliness. De Jong Gierveld and colleagues recommend categorising scores of 2 to 4 on this scale as moderately lonely and 5 to 6 as severely lonely [28]. Other inclusion criteria have been kept deliberately broad, although we will prioritise service users currently receiving support from several disciplines (e.g. care coordination, psychology and medical review), to help us explore whether Community Navigation is a useful addition to multi-disciplinary care from a secondary mental health service.

Inclusion criteria:

- Currently on the caseload of a secondary mental health service for people with depression or anxiety
- Aged 18 years or older
- Score at least 2 on the six-item De Jong Gierveld Loneliness Scale [27] at initial screening

Exclusion criteria:

- People who do not have capacity to consent to participate
- People who pose a risk of harm to others (as judged by their care coordinator) such that meetings with a researcher or Community Navigator are not recommended
- People who are unable to communicate in English. Resources were not available to deliver this early stage intervention in other languages
- People who are currently an inpatient at a mental health or general hospital or using mental health crisis services

An overview of the recruitment process can be seen in the flow diagram (Fig. 1). Service users will be screened for eligibility and approached initially by clinical staff from the participating team that supports them, who will explain the study briefly and ask if they are willing to be contacted by a researcher. A study researcher will then contact potential participants to explain what the study involves, answer any questions, and conduct the screening questionnaire. Once the study researcher has established that people are eligible to take part, potential participants will be sent a study information sheet. The researcher will make contact again to check that the participant has understood the information sheet and has continued capacity to consent. Written consent to participate will be obtained using a Consent Form (Additional file 2) at a face-to-face meeting prior to data collection and randomisation.

### Randomisation

Participants will be randomised to a treatment group (*n* = 30) or a control group (*n* = 10). Randomisation will be stratified by study site and will be 3:1 for intervention to control using block randomisation. An independent statistician in UCL Division of Psychiatry will generate the allocation sequence using Stata, which will be shared with the chief investigator. It will be concealed from the study researcher, who will be blind when recruiting participants and collecting baseline data. Once participants have completed the baseline questionnaires, the researcher will contact the chief investigator to ascertain the outcome of their randomisation. Due to limitations in the researcher resources available, participants’ allocations will not be concealed from the research team. The study researcher will contact participants to let them know the outcome of randomisation, send those in the control group information on local resources, and, for those in the intervention group, inform clinical supervisors that they are ready for allocation to a Community Navigator.

### The intervention

#### Development

Intervention development includes modelling, preliminary testing and a feasibility trial of the programme, in accordance with guidance for developing a complex intervention [25]. The intervention is being designed collaboratively using principles of coproduction [29]. We have a working group including people with expertise from: experience of managing depression, anxiety, and loneliness personally (experts by experience); working in services supporting people with complex depression and anxiety (practitioners); researching social interventions to address loneliness and mental health problems (research team). The working group is equally balanced between these groups, with junior researchers and experts by experience taking chairing roles in meetings to avoid a hierarchical atmosphere. The working group are drawing on several sources of information to develop the intervention:

- Current academic literature presented to the working group by the research team. As part of a related review, a scoping review of the academic literature was performed to identify existing interventions targeting loneliness and the evidence base for these interventions [19]. Academic literature describing models of loneliness and the theoretical basis for interventions targeting loneliness [30] was also reviewed and presented to the working group
- Consultations carried out by the research team within the two local services (CDAT and CCT) with current service users and clinicians to understand their views on loneliness and what intervention might be useful. This information was presented to the working group
- Expert speakers invited to the working group to present and facilitate discussion including Wellbeing Enterprises CIC [31] and Peter Bates from the National Development Team for Inclusion [32]
- Meetings by the research team with the Groups 4 Health programme team [33] to explore a potential group intervention for our Community Navigation programme

The working group of 12 people met regularly in the set-up phase to develop the intervention. This group will continue to meet to guide intervention development, refine a Theory of Change for the intervention, advise on trial processes and perform analysis of qualitative data. Experts by experience’s contribution to this working group constitutes paid involvement, offered because of their expertise through experience, and is reimbursed in accordance with national guidelines [34].

The intervention was refined in response to the experience of, and feedback from, preliminary testing. In preliminary testing, five participants were recruited from each study site (total *n* = 10) and received support from a Community Navigator over a period of 6 months. Qualitative interviews were conducted with all available participants (*n* = 6) and the Community Navigators (*n* = 3) by interviewers with personal experience of depression and anxiety. Interview transcripts were analysed by study researchers, with input from the working group, with the main aim of identifying any modifications needing to be made to the programme for the feasibility trial. These modifications have been discussed within the study team and working group and the current version of the trial protocol was approved by the Research Ethics Committee.

#### Theoretical basis

The assumption that increased social connection can reduce loneliness and thus reduce depression has both theoretical and empirical support. Loneliness has been demonstrated to predict poor recovery from both depression and anxiety [18], while a recent Australian study provides promising preliminary evidence that a socially-focused group programme reduced loneliness and subsequently depression in a population of young adults with mild mood disorders [35].

According to the Social Identity Approach to health [36, 37], enhanced identification as a member of social groups and communities may lead to improved access to social support, a greater sense of control and enhanced self-esteem. Community Navigators will help participants to expand their awareness of opportunities for social contact. They will adopt a strengths-based, solution-focused approach [38] which will encourage participants to focus on what they can do. Community Navigators will also seek to normalise setbacks participants encounter with increasing social connections. Group sessions may serve to normalise worries about social interaction and provide inspiring examples of positive behaviour change. In these ways, participants’ hope, confidence and sense of self-efficacy may be increased. Ideally, a ‘virtuous circle’ will be initiated, where increased social interaction fosters more positive thinking, which prompts and enables further social interaction.

In this intervention, up to ten sessions are offered, rather than the briefer programmes of socially focused support typically offered in primary care settings [20]. This recognises that the process of engagement, agreeing manageable goals and problem-solving barriers or setbacks may all take longer with a severe and enduringly ill client group. Effective engagement with participants will be enabled by building flexibility into the programme, to maximise its fit with participants’ needs and preferences.

#### Principles of Community Navigation

There are five principles which are central to the way that Community Navigators work with people:

- Socially focused – Community Navigators will focus exclusively on support to enhance an individual’s social world, working with people to feel more engaged and connected to other people, activities, and their community
- Asset-based – Community Navigators are champions of the community. They will continually seek to develop their knowledge of the local community and use this to connect individuals to resources that help to promote and sustain wellbeing
- Solution-focused [38] – together with people they are supporting, Community Navigators will seek to identify the next step towards achieving their goals. The approach is future-focused, looking for positive solutions using the person’s strengths and resources rather than concentrating on the past or what is preventing the person from moving forward [38]
- Person-centred – support will be individualised, focussing on the needs, goals, and preferences of the individual at each point in their recovery. Community Navigators will work collaboratively ‘with’ the person rather than doing things ‘to’ or ‘for’ the person
- Non-directive – Community Navigators will have their own ideas, goals and agenda, but these will be secondary to those of the person they are supporting, who will direct the pace and direction of their journey together

Community Navigators will also use the GROW model of coaching when supporting people [39]. This involves identifying a ‘Goal’ and then exploring the present ‘Reality’. Next, people are encouraged to explore all of their ‘Options’ and finally commit to an action in the ‘Way forward’. This approach will be client-led and will allow participants to identify their own goals and plan [39]. It is conceptually congruent with the solution-focussed approach.

#### Intervention outline

Those receiving the intervention (*n* = 30) will be offered up to ten, hour-long meetings with a Community Navigator and access to up to three group sessions over a 6-month period alongside their standard care. Participants can have as many meetings as they want over the 6 months, up to a total of ten, and these meetings can be scheduled whenever they are most helpful within the 6 months. Each participant may use a budget of up to £100 on goals agreed with their Community Navigator to facilitate access to and participation in activities that provide opportunities for developing social connections. Meetings may take place in participants’ homes, community spaces or NHS premises, as the participant prefers.

The Community Navigation intervention will comprise three main components. Firstly, Community Navigators will build up rapport through discussing the person’s social network and interests. A social network mapping tool will be used to identify the people, places and activities that are important to the person. Items will be plotted in terms of importance and may be current, previous or potential new connections that the person would like to form. The aim is to collaboratively identify the person’s current interests and social connections, potential areas for new activity or social contact and how existing connections could be strengthened.

This social network mapping tool is bespoke to Community Navigation, and will be co-developed within the working group with input from the Community Navigators based on people’s experiences. It will be informed by existing interventions to map social systems and activity and primarily based on a similar mapping process [40]. This existing process involves getting people to generate the names of people that they know, places that they go to in their community, and the sort of activities that they do regularly. People are then asked to place all of the names generated on a ‘map’ consisting of concentric circles, with items they feel closest to in the inner circles [40]. Social network reviews may be informed by Wellbeing Reviews, a process developed by Wellbeing Enterprises CIC and reported in the Campaign to End Loneliness ‘Promising Approaches’ bulletin [13]. This involves identifying social issues that may be causing or exacerbating health problems. Community Navigators may also use the ‘self-aspect pie’ to explore people’s social networks. This is a tool from the Groups 4 Health programme, which prompts people to reflect on and visually present their self-concept (the important aspects of themselves) and consider whether it is well represented by their social identity [35].

Secondly, Community Navigators will provide support to develop and use an action plan to increase connectedness. This action plan will involve setting goals around connecting, reconnecting, exploring opportunities in the community, and joining in with new groups or activities. The plan will be based on ‘SMART’ principles, meaning that goals should be specific, measurable, attainable, realistic and timely. Creating this plan will involve identifying strengths and resources that people currently have which may help them achieve their goals. Community Navigators will help participants to break goals down into a series of steps, so that progress may be made, even towards challenging or long-term goals. Community Navigators will use their local knowledge of leisure activities, cultural and social support groups, and wellbeing support to help people develop this plan. The Community Navigators’ role includes time to ‘asset map’ local communities to identify useful groups and social resources in general and in relation to goals agreed with individual participants.

Support to use this action plan will be person centred and include: providing information about available activities and sources of support locally; practical help to access activity (e.g. planning travel routes or accompanying the participant to a new social group); access to financial support from a budget of £100 per participant; reinforcing strengths and successes; problem-solving challenges and setbacks; and providing encouragement and emotional support to increase social connectedness. Community Navigators can also utilise exercises from the Groups 4 Health programme to encourage people to focus on beneficial social activities and to develop reciprocity and give more to others in social interactions, potentially eliciting more positive responses [35].

Finally, participants will be invited to attend up to three sessions to meet co-participants, discuss the programme’s aims and their progress, and share information about helpful local resources and experiences. One session will be early in participants’ support from their Community Navigator, during the first half of the 6-month intervention period, and one towards the end of the programme. A third session may be added, depending on the enthusiasm for this in the group. Attendance is optional but Community Navigators will encourage and support all participants to join in where possible. Sessions will last approximately 2 h, with a break, and will be facilitated by the Community Navigators. Each meeting will have a loose agenda tailored to the needs of those taking part to achieve maximum impact. The group will follow the principles of the Community Navigator programme with a socially focused, person-centred approach.

The Community Navigation programme is a social intervention focussed on increasing social contact and connection. Community Navigators will be dissuaded from helping participants with other problems (e.g. medication management, housing, employment, debts or welfare benefits), for which they may not have the time or clinical skills, and for which the participant may already be receiving support. Instead, Community Navigators will be encouraged to signpost participants back to the involved clinical team for help with these issues.

#### The Community Navigators

Community Navigators will be embedded within participating services in Camden and Islington NHS Foundation Trust and Barnet, Enfield and Haringey Mental Health NHS Trust. They have been recruited specifically to this role and do not need a professional mental health qualification. Lived experience of mental health problems constitutes relevant experience, but is not essential. Excellent interpersonal skills, sensitivity to and understanding of mental health difficulties, local knowledge, and awareness of assets in the community are essential requirements for the role. In this feasibility trial, three Community Navigators will be working with participants across Barnet, Camden, and Islington. Recruitment to these posts involved the study working group, who developed exercises to assess applicants’ rapport-building skills and awareness of community assets. Community Navigators will be supported to fulfil their role through training and supervision.

##### Training

A five-day initial training programme provides instruction and practice in key activities of the role: community resource-finding; mapping people’s social worlds; developing a personal social connections plan; using a solution-focused approach [38]; and the GROW coaching model [39]. Training is experiential in nature using group discussion and scenarios. Trainers with lived experience of depression or anxiety will help Navigators to role play tasks offering realistic practice and valid feedback. Practitioners from involved clinical services will also provide training about local service structures and available crisis support, the nature of the participant group, and guidance on how to respond to safety concerns regarding participants or urgent clinical needs. The Community Navigators will also learn through discussion and direct observation of experienced staff employed in social navigation roles at Wellbeing Enterprises CIC and Bromley-By-Bow Centre social prescribing projects.

##### Supervision

Regular group supervision (monthly at each of the two services in this trial) will provide support with delivering the study activities and address issues or challenges in working with specific participants. Supervisors are mental health practitioners from participating services who know the study participants, bring clinical expertise and local service knowledge, and who are familiar with and supportive of the structure and aims of the Community Navigator programme. The Community Navigators will also have immediate access at all times to clinical staff in the event of any immediate concerns about participants’ safety or wellbeing.

### The control group

Participants in the control group (*n* = 10) will be offered written information about community resources and activities within their area. Participants will otherwise receive standard care, unaffected by their participation in the study. This was chosen as a comparator to demonstrate whether support from a Community Navigator is effective over and above usual care received from multidisciplinary teams with enhanced knowledge of resources generally available in the local area.

### Outcome measures

#### Procedure

All participants in the feasibility trial will be asked to complete self-report questionnaires at baseline and at the six-month end of intervention follow-up, through a structured interview with a study researcher. Additionally, 20 participants receiving the intervention, the three Community Navigators and ten other stakeholders (the Community Navigators’ supervisors, and other involved mental health team staff and participants’ involved friends or family) will be asked to take part in a qualitative interview following the intervention. Interviews will explore acceptability of the community navigator programme and views on its impact from several perspectives. This is described in the SPIRIT flow diagram (Fig. 1) and the SPIRIT trial schedule (Table 1).

Information about all study assessments is provided in the participant information sheets, and consent to collect data and contact participants about follow-up is included in consent forms. Written consent from participants will be confirmed before the 6 month follow-up questionnaires are completed. A separate information sheet and written consent from participants will be provided for qualitative interviews. Participants will be offered a £20 gift in cash to thank them for their time at three time-points (upon completion of each of the baseline and follow-up questionnaires and the qualitative interview).

#### Measures

A number of potential primary and secondary outcome measures for a future definitive RCT will be included. These self-report questionnaires will be assessed for response and completeness at baseline and six-month follow-up. The battery of measures for use in the study was reviewed and shortened following feedback from preliminary testing and discussion with the working group. Data collection will begin by asking participants for information on their socio-demographic characteristics including age, gender, ethnicity, marital status, living arrangements, accommodation, and employment. Participants will then be asked to complete the following validated outcome measures, all of which provide data for analysis in continuous form:

- The De Jong Gierveld Loneliness Scale is an 11-item, self-report measure of loneliness, yielding a total score and subscale scores for social and emotional loneliness [41].
- The Lubben Social Network Scale is a six-item self-report measure assessing quantity and quality of contact with family and friends [42].
- The Resource Generator UK is a 27-item measure of perceived access to social capital [43].
- The Time Budget Diary is a retrospective self-report measure of activity over the previous week [44]. Additional questions have been added to this measure so that participants are asked whether activities were done with others or alone and, if with others, whether this was online, phone or face-to-face. These additions will allow us to distinguish activity involving social contact from other activity.
- The Warwick-Edinburgh Mental Well-being Scale is a 14-item self-report scale of mental wellbeing [45].
- The Patient Health Questionnaire (PHQ-9)is a nine-item self-report measure of depression [46].
- The Generalized Anxiety Disorder Questionnaire is a seven-item self-report measure of anxiety [47].
- The EQ-5D-5 L is a five-item self-report health outcome measure [48].
- The Recovering Quality of Life Questionnaire is a 10-item self-report measure of quality of life developed for use across all mental health populations [49].

These measures were chosen by the working group after extensive discussion balancing data collection needs and respondent burden. They aim to capture dimensions which support from a Community Navigator may improve including mental health symptoms (depression, anxiety), social outcomes (loneliness, social network, social capital), day-to-day activity and general wellbeing (mental wellbeing, quality of life).

We will seek information from Trust Informatics teams regarding participants’ current diagnosis, care cluster, attended and missed face-to-face appointments with their mental health team, use of other community mental health services, admission to acute care, days in inpatient care, and use of the Mental Health Act. These records will be sought for the six months prior to baseline and at six months’ follow-up for the intervention period. Participants’ use of social care services will also be sought from local council social care records at the same time points.

#### Qualitative interviews

At or towards the end of their sessions with their Community Navigator (after a minimum of five sessions have been received, or the participant has elected to discontinue meetings with their Community Navigator), a researcher will contact participants for a qualitative interview. Members of the team who will be carrying out the interviews with participants have personal experience of depression and anxiety, and topic guides for all qualitative work will be coproduced with the study working group. These interviews will explore people’s experiences of the programme, including:

- The content of sessions with Community Navigators
- The impact of being part of the programme
- How the programme proved helpful
- Challenges around being part of the programme
- Suggested improvements to the programme

Qualitative interviews will be conducted with participants (*n* = 20), Community Navigators (*n* = 3) and up to ten other stakeholders. These could include the Community Navigators’ supervisors, other clinicians, social care or voluntary sector staff such as peer support workers, and family and friends nominated by participants as important to them in utilising the support. We will purposively sample 20 participants receiving the intervention for a qualitative interview as far as possible to ensure representation from all services and participants with a range of demographic and clinical characteristics, including those who completed the programme of Community Navigation and those who discontinued the intervention early.

These interviews will be used to refine a Theory of Change for this programme. This is a model which outlines the processes by which the programme may have an effect [50]. The Theory of Change will be built around the following components:

- Assumptions - the context in which the programme is taking place and how it will work
- Inputs - the resources put in to deliver the programme
- Activities - the content of the programme
- Enablers - factors facilitating the delivery of programme outcomes
- Intermediate outcomes – the shorter-term changes or impacts of the programme
- Final goals – the broader-longer term aims of the programme

#### Process recording

To facilitate examination of the intervention content, Community Navigators will complete session logs following each session with a participant, detailing the location of the meeting and its content (selecting from a list of planned types of support). Feedback will be sought from intervention-arm participants for two of their Community Navigation sessions. Participants will be contacted as soon as possible (ideally within 3 days) following a randomly selected session and asked questions over the phone by a study researcher. Questions, taken from a standard feedback form, will cover the location of the meeting, what types of support were provided (with the same options as in Community Navigators’ session logs), and a rating of how they found the session on a five-point scale (from very good to very poor). The aim is to have data spanning all ten sessions without having to ask participants for feedback after each session.

### Data analysis

Factors relating to the acceptability of the intervention and the feasibility of trial procedures, and completion rates for potential primary and secondary outcomes in a definitive RCT will be reported.

Feasibility will be assessed by:

1. Recruitment duration; the time period from recruitment of the first trial participant to meeting the trial recruitment target (40 participants).
2. Recruitment; the number of participants screened, the number of those screened who are eligible, and the number of eligible participants who consent to participate in the study by four months.
3. Attrition; the number of participants who consent to participate that remain in the study until the end of follow up at six months.
4. Intervention take-up rate for those in the intervention arm: the proportion of participants who met their Community Navigator
5. Implementation of the intervention: the proportion of participants that maintained engagement with a Community Navigator and the number of sessions of support provided. A minimum threshold of at least three meetings with a Community Navigator has been set to represent treatment as per protocol.
6. Number of adverse events recorded in each study arm until the end of follow up at six months.

#### Quantitative measures

We will report rates of missing data and summary statistics for all outcomes, both overall and by randomised group, at baseline and at follow-up. This trial will be too small to make clear inferences about observed differences between groups. However, for two candidate primary outcome variables for a future definitive trial: loneliness (measured using the 11-item De Jong Gierveld Loneliness Scale [41]) and depression (measured with the PHQ-9 [46]), we will calculate an effect size (non-standardised mean difference between groups) with confidence intervals, to assess the potential for the intervention to affect outcomes.

#### Qualitative interviews

Data will be analysed using a thematic analysis approach [51], with involvement from members of the working group. We will hold analysis meetings to review transcripts, develop coding frames, and review themes. Some of those who will be involved in qualitative interview analysis have personal experience of depression and anxiety. Expertise by experience will explicitly be drawn upon in the analysis process, following the McPin Foundation approach to peer research [52]. The research will thus benefit from combining research skills and lived experience in the analysis and synthesis of data [53]. Analysis will focus on understanding people’s experience of the intervention, what could be improved about it and informing the Theory of Change.

#### Process recording

Descriptive information from Community Navigators’ and participants’ process records will be reported to describe the content of the intervention. For sessions where both the Community Navigator and participant have completed session logs, levels of agreement on the types of activity undertaken in the session will be assessed and inter-rater reliability calculated using Cohen’s kappa [54]. This will provide an indication of the accuracy of Community Navigators’ reports of session content and demonstrate whether they view session contents differently to participants. Summary statistics of participants’ ratings of the quality of sessions with Community Navigators will also be reported.

### Data management

All participant consent forms and quantitative study data will be stored at UCL. Consent forms identifying participants will be stored separately from case report forms, which will not bear the participant’s name or other personal identifiable data. All paper forms will be kept in locked cabinets in secure offices. Study researchers will develop and manage a secure database for all quantitative study data using SPSS software [55] on the secure IT network at University College London. Qualitative audio files and transcripts will be kept similarly securely at McPin offices for the duration of the study. Anonymised electronic copies of qualitative transcripts will be stored using QSR International’s NVivo 10 qualitative data analysis software [56] at the McPin Foundation. After the study, all data will be archived securely at UCL.

### Data monitoring

The study sponsors, University College London, act as guarantors for the trial, including insurance and indemnity arrangements, and are responsible for overseeing and auditing trial conduct, through the UCL/UCLH Joint Research Office. Any proposed changes to the trial protocol during the study will be agreed by the study team and submitted for approval to the research ethics committee. Protocol modifications will then be communicated to the sponsor, site principle investigators, participating NHS trusts and trial participants via email or telephone. Service users, staff and other stakeholders providing data for the trial will all provide informed consent to take part, using ethically approved procedures.

The trial will be run day-to-day by a study management group including the Chief Investigators and other senior academics and clinicians. The study management group will meet approximately six times per year and will send updates to a Principal Investigator at each site. Independent advice to the study management group and oversight of the study is provided by a trial steering committee, which is independent of the sponsor and will meet at least annually during the trial. The steering committee comprises senior academics, a statistician, and a service user representative. A data monitoring committee (DMC) is not planned for this small feasibility trial, but the trial steering committee will advise if any role for a separate DMC is indicated during the trial. No interim analyses are planned, and no stopping criteria are pre-set.

The study researcher will screen for serious adverse events through review of intervention session logs, monthly updates from the clinical supervisors, and review of patient records by clinicians in participating services. Any serious adverse events reported to the study team will be reviewed by the study Chief Investigator and by the chair of the trial steering committee as an independent reviewer. Any adverse events assessed as study-related will be reported, with the trial steering committee chair’s recommendation, to the study sponsor.

## Discussion

### Strengths and limitations

This intervention is being developed by a working group of 12 people including experts by experience, practitioners and members of the research team. Service users and clinicians from the mental health teams involved in the study, as well as experts delivering similar programmes, are being consulted. This increases the likelihood that this intervention is relevant to the study population and works within these clinical settings [57]. The literature also suggests that coproduction makes sustainable change and translation of knowledge into practice more likely [58, 59].

Throughout this study, Medical Research Council guidance on developing and evaluating complex interventions [25] is being followed in a robust manner. Before starting this feasibility trial, Community Navigation was piloted with ten service users to test and refine the intervention and procedures for recruitment and outcomes data collection, enhancing their acceptability to participants, clinicians and researchers. Additionally, preliminary testing allowed us to provide further training for the Community Navigators and support consistent delivery of the intervention.

The use of qualitative interviews with service users, their friends and family, clinicians, and Community Navigators will inform us about people’s experiences. This will help establish the acceptability and feasibility of the programme, and inform any further refinements to the intervention needed before a future definitive trial. The interviews will also be used to refine a Theory of Change, modelling the resources needed to deliver the intervention, its content and the outcomes achieved. Qualitative interviews will provide insight into people’s experiences, which may reveal the mechanisms through which the support has its effect, and what factors need to be in place to achieve successful outcomes.

This feasibility trial will run across two NHS sites including affluent areas and areas of high deprivation as well as ethnically diverse populations. Due to limitations in resources, we are only able to include service users who can communicate in English. This limits the generalizability of our findings to English-speakers within these services. However, this is still a diverse group of service users. Except for this requirement, we have kept the inclusion and exclusion criteria broad so that most users of the participating services will be eligible to take part. This will maximise learning about the extent to which Community Navigation is generally feasible and acceptable in addition to standard care from secondary mental health services.

As researchers will have ongoing contact with clinical teams, Community Navigators and participants throughout the intervention, it will not be possible for researchers to be blinded during outcomes data collection. However, outcome measures will be self-administered so should not be influenced by researcher bias. In a future definitive RCT, with more resources available, it would be possible for researchers to be blinded, but this procedure will not be tested in this trial.

This intervention takes one approach to targeting loneliness. There are other components of loneliness which we are not attempting to tackle. For example, people who feel lonely may have cognitive biases, such as negative evaluations of others, and a lack of interpersonal trust [60]. Alternative interventions for loneliness try to change people’s cognitions about social relationships, for example cognitive ‘reframing’ of loneliness to increase perceived control over reducing it [61]. Community navigation does not include a cognitive component, or other approaches such as social skills training and wider community changes. With current evidence, it is unclear which approach to reducing loneliness and increasing people’s community connections may be most effective [19].

### Research implications

Although social interventions such as Community Navigation are advocated in policy, and have been reported positively, there is little evidence to date regarding their effectiveness [24]. The appropriateness of using such interventions (most commonly provided in primary care) for a population using specialist mental health services with enduring mental health problems and complex needs is also unclear from current research. In this feasibility trial, we attempt to address these issues and provide preliminary evidence of the acceptability of this support.

Evidence regarding the feasibility of Community Navigation should provide a basis for future research. If this feasibility trial of the Community Navigator programme yields promising results, it will provide a clearly defined structure and set of resources that can be definitively tested in a large-scale RCT. In addition, this study should reveal barriers and facilitators to implementing this social intervention within an RCT. This should enable us to implement the most effective recruitment strategy, develop clear trial procedures, consider key roles of clinical staff in the study, and address people’s willingness to participate in this type of intervention. Our findings may have broader implications to inform randomised trials of social interventions within mental health services and in other areas.

### Implications for policy and practice

Loneliness can negatively influence the physical, psychological and social wellbeing of individuals. Interventions which alleviate loneliness are beneficial in their own right and hold promise in improving people’s mental health, as well as having wider benefits for health outcomes and health service resources. If successful, this feasibility study will provide a clearly manualised social intervention designed to reduce loneliness, with evidence of acceptability and feasibility for use in mental health settings. As UK policy already advocates this kind of social intervention, and related programmes are already running, this would be immediately useful in guiding commissioning and delivery. It would provide a manualised intervention ready for delivery in clinical settings. This feasibility trial will also inform and support a future larger RCT of Community Navigation, which could establish the effectiveness of this type of support and potential cost savings, calculated through an economic analysis.

## Trial status

This is protocol version 2 (09/02/2017) which received ethical approval on 14/03/2017 and Health Research Authority approval on 23/03/17. Participant recruitment began on 24/04/2017, is ongoing at the time this paper was submitted, and is expected to be complete by the end of August 2017.

## Additional files

Additional file 1: Community Navigator Feasibility Trial – SPIRIT Reporting Checklist. (DOCX 59 kb)

Additional file 2: Community Navigator Feasibility Trial – Participant Consent Form. (DOCX 68 kb)

## Abbreviations

Barnet CCT: Barnet Complex Care Team
Camden and Islington CDAT Team: Camden and Islington Complex Depression Anxiety and Trauma Team
CIC: Community Interest Company
DMC: Data Monitoring and Ethics Committee
NIHR SSCR: National Institute for Health Research School for Social Care Research
RCT: Randomised Controlled Trial
SPIRIT: Standard Protocol Items: Recommendations for Interventional Trials
UCL: University College London
