# Supplementary material for: The Community Navigator Study: a feasibility randomised controlled trial of an intervention to increase community connections and reduce loneliness for people with complex anxiety or depression
Source: Trials. 2017 Oct 23;18:493. doi: 10.1186/s13063-017-2226-7 (PMC5654042; doi:10.1186/s13063-017-2226-7)
Supplement: Supplementary file 2 — Community Navigator Feasibility Trial – Participant Consent Form. (DOCX 68 kb) [file 13063_2017_2226_MOESM2_ESM.docx]

**UCL Division OF PSYCHIATRY**

faculty of brain sciences

BLOOMSBURY campus

IRAS project ID: 200767

**CONSENT FORM FOR PARTICIPATION IN THE COMMUNITY NAVIGATOR STUDY FEASIBILITY TRIAL**

Version 2. 09/02/2017.

**Study Title: Community Navigator Study. Evaluation of an intervention to increase community connections and reduce loneliness for people with complex anxiety or depression.**

Principal Investigators: Professor Sonia Johnson and Dr Bryn Lloyd Evans (University College London, UCL)

| 1. I have read and understood the study information sheet Version 2 dated 09.02.17. I have had the opportunity to ask questions about the study. |  |
| --- | --- |
| 1. I understand that my participation is voluntary and that I can withdraw at any time, without giving any reason, and without it affecting the support provided to me by services. |  |
| 1. I understand that the service which supports me and my GP will both be informed that I am taking part in the study. |  |
| 1. I agree that a researcher may contact me at subsequent points during this year, to see whether I am willing to take part in other data collection activities. I am aware that when they contact me, I can decide whether to take part or not. |  |
| 1. I understand that if I receive support from a Community Navigator, a record of our meetings will be kept in my patient notes. I agree that the research team can also receive a version of these notes, in which I will not be identified by name, and from which any personal information will have been removed. |  |
| 1. I consent to the research team having access to information about my diagnosis and use of mental health and social services from my NHS and social services electronic patient records. |  |
| 1. I consent to the information collected about me for this study being stored securely at University College London (UCL). |  |
| 1. I understand that relevant sections of my medical notes and data collected during the study may be looked at by individuals from regulatory authorities and/or the NHS Trust, where it is relevant to my taking part in the research. I give permission for these individuals to have access to my records. |  |
| 1. I understand that I will be offered a £20 gift in cash for my participation in the research interview, once I have taken part in it. |  |
| 1. I agree to take part in the study. |  |

My preferred contact details

Address:

Phone number(s):

E-mail address:

Preferred method of contact:

Phone

E-mail

Letter

I would like a copy of a report with the study findings when the study is over:

Yes

No

________________________ ________________ _____________________

Name of participant Date Signature

________________________ ________________ _____________________

Name of researcher Date Signature
